# X-Ray Therapeutics

**Published:** 1906-12

**Authors:** James Taylor

**Affiliations:** Medical Officer to the X-ray Department, Royal Infirmary, Bristol


"*Scire est nescire, nisi id me*
*Scire alius sciret.*"

DECEMBER, 1906.

INDEX THROUGH

### X-RAY THERAPEUTICS.

The Presidential Address, delivered on October 10th, 1906, at the opening of the Thirty-third Session of the Bristol Medico-Chirurgical Society.

BY

JAMES TAYLOR, M.R.C.S.

*Medical Officer to the X-ray Department, Royal Infirmary, Bristol.*

MY first duty is to return you my best thanks for the honour that you have done me in electing me your President for the ensuing year, an honour that is perhaps the highest a medical man can hope to attain in this city. Once again your choice of President has fallen on one of the rank and file of the profession, for although it has been my pride and privilege to have been connected with the staff of the Infirmary in a subordinate position for the last few years, still all my medical career has been passed as a general practitioner. As such I should like to express my gratitude to this Society for help I have received in my professional work. In these days of increased knowledge and great activity in medical research it becomes increasingly difficult for the man engaged in the arduous work of general practice to keep himself at all *au fait*

with recent medical thought. Take, for example, the various "ostomies" and "ectomies" of modern surgery, also the bewildering vocabulary that has come into vogue in connection with diseases of the blood—toxins, antitoxins, etc. What an assistance it is to a man who has not the good fortune to be attached to a large hospital, where all these things are, so to say, in the air, to come to our meetings and hear all the latest ideas on these and kindred subjects brought forward and discussed. Speaking for myself, I have found these meetings of the greatest service to me, and I cannot help thinking that many of my brother practitioners, though they may be handicapped by having evening professional engagements, are neglecting their best interests in not making more strenuous efforts to attend the meetings of this Society.

It so happens that this year of grace 1906 is the thirtieth anniversary of my entry into medical practice, and it cannot be but that under the circumstances one's thoughts should be somewhat retrospective. It seems to me that this period of thirty years is one of the most, if not *the* most, momentous in medical history, for it is hardly too much to say that during this time, thanks to the epoch-making researches of Lister, the whole of medical thought has been revolutionised. It is only by looking back and trying to remember the state of medical science at the time when I first began my medical studies that one realises the enormous advances that have been made in every branch of medicine during the last three decades. This advance is perhaps most marked in surgery. Contrast the surgical procedures of thirty years ago with now. Then one day a week, or at most two, were sufficient for the surgical operations of a large hospital; now several operating theatres are required in a well-equipped hospital, these being in constant use every day of the week. Then again, the whole of abdominal surgery as we know it at the present day has been worked out; and we in Bristol ever remember with pride that one of the pioneers in this great work did his work here in our midst, and that it is largely owing to the labours of Greig Smith, whose death in the prime of his manhood we still continually deplore, that abdominal surgery stands where it does to-day.

But if the advance in surgery has been so great, the advance in medicine, though perhaps not so apparent to the proverbial man in the street, has been equally great, and this too is due to the revolution in thought caused by Lister's discoveries. His theory of "germs" naturally led observers to try and demonstrate such germs, one of the earliest discoveries being that of the discovery of the tubercle bacillus by Koch, since which date one may say that the whole modern science of bacteriology has arisen, and has entirely altered the conception of disease.

Long before this time attempts had been made to discover the parasitic cause of disease, and amongst the most notable of these attempts were those of the first President of our Society, Dr. Brittan, in conjunction with Dr. J. G. Swayne, who tried to discover the cause of cholera by microscopically examining the air of the Bristol Bridewell during the cholera epidemic of 1848: when one looks at the drawings of the latter, one cannot help thinking that the discovery of the cholera bacillus was very near being made in Bristol, instead of being "made in Germany."

Of course these discoveries have led to a more exact treatment of disease; and as in the case of diphtheria the study of the life-history of the Löeffler bacillus has led to the treatment by antitoxin, and thus shorn the disease of half its terrors, so in other diseases doubtless the antitoxin treatment, in one form or another, will become still more perfected, for as yet, I take it, we are only on the threshold of bacteriological knowledge.

Treatment of disease leads me to the subject to which I wish particularly to direct your attention this evening, and that is the treatment of diseased conditions by the X-rays.

It is now some ten years ago that Röntgen gave to the world his discovery of what he happily termed the "X-rays," and thus opened a new field of inquiry to the physicist, and at the same time gave to surgery and medicine a new power in diagnosis and treatment. It is needless to dwell on the aid the X-rays are to the surgeon, and to the physician as well in diagnosis, but I would rather dwell on their use as a method of treatment.

On their first introduction, with the imperfect apparatus at the command of the operator, very long exposure to the rays was necessary to get an image on the photographic plate, and, as we know now, it was not to be wondered at that very soon marked destructive action on the skin exposed to the rays was observed, a dermatitis being set up which was followed by extensive ulceration of a most obstinate character. Early workers at radiography were amongst the worst sufferers from this action of the rays, owing to their testing the penetrating power of their tubes by holding their hands between the focus-tube and the screen, and there are many men going about to-day with an incurable condition of the skin and nails of their hands due to this cause.

The skin thus damaged was soon examined microscopically by many observers, and the main conclusion from their investigations was that it was the cellular element of the tissues that was found to be affected. Thus Scholtz,[1] quoted by Pusey and Caldwell,[2] after many experiments on the skin of animals, says: "(1) X-rays influence especially or exclusively the cellular elements of the skin. These are inflamed primarily, and undergo a slow degeneration, whilst the connective tissue, the elastic tissue, musculature, and cartilage are changed only in a slight degree, and suffer only secondarily as a result of the cellular degeneration and the inflammatory reaction consequent to it. (2) The degeneration affects the epithelial cells in the highest degree, and to a less extent the cells of the glands, the vessels, and muscular and connective tissues. (3) The degenerative appearances are of various kinds, and affect both the protoplasm and the nuclei of the cells. (4) As soon as the degenerative process has reached a certain point, an inflammatory reaction appears, which manifests itself in a marked dilatation of the vessels, with gathering leucocytes and marked emigration of the blood corpuscles. When there is greater cell-degeneration as a result of stronger exposure, collections of leucocytes press into a mass of degenerated cells and accomplish their further destruction.

Various investigations have been made as to the effect of

[1] *Arch. f. Dermat. u. Syph.*, 1902, lix. 241.

[2] *The Röntgen Rays in Therapeutics and Diagnosis*, 1903, p. 258.

the X-rays on all kinds of animal matter. Amongst the most interesting are those of Bordier and Galimand,[1] who subjected hen's eggs to the action of X-rays with the object of seeing what effect they would have on development. They placed a certain number of eggs in an incubator, half of which they exposed from time to time to the rays, and the other half they kept as controls. These latter went on in the incubator to perfect development, but not one of the rayed eggs showed a trace of embryonic formation, and in addition to this, the contents had altered in appearance: the white and the yolk were distinct, the white being more fluid than that of a fresh egg, and the yellow more granular than normal.

Again, Higham Cooper[2] having noticed symptoms of toxæmia in patients he had treated for leukæmia, suggests that the symptoms were set up owing to the decomposition of lecithin by the rays, and quotes Werner, who found that lecithin when exposed to X-rays undergoes a kind of decomposition. He also quotes Halliburton, who states that in the ordinary metabolic processes lecithin is split up into stearic and glycero-phosphoric acids and choline. The presence of the latter in the blood would of course explain any toxæmic symptoms, but in addition to this we know that the nervous tissue is one of the tissues easily acted on by the rays, and also that it contains a large quantity of lecithin. From this he suggests that the changes noticed as the result of treatment by X-rays is after all a trophic one due to the destruction of the nervous tissue, the lecithin being broken up in the manner described.

As regards the mode of action of the X-rays, the same author gives a very suggestive experiment he has made with a photographic plate, tending to show that animal matter is necessary to the photographic result of the X-rays.[3] Thinking possibly that the gelatine in the silver emulsion used for coating the ordinary photographic plate may have something to do with the photographic impression made by the rays, he tried the effect of exposing a plate coated with collodion instead of gelatine, and found that the plate was practically unaffected by the radiation from the tube.

[1] *Med. Electrol. and Radiol.*, vi. 161. [2] *Ibid.*, vii. 3. [3] *Loc. cit.*

Now if these investigations of Cooper, Bordier, Galimand and others are substantiated, it seems to me that a great advance has been made in placing the action of radio-active bodies on a scientific and sure footing. As to how these changes in animal tissues are produced has been, and for the matter of that still is, a vexed question. The most probable explanation is that of Sir Oliver Lodge,[1] who considers that the destructive effects of the rays are "a secondary effect, and are due to ultra-violet light . . . and to the chemical or ionising action of the rays upon the tissues or upon air in immediate contact with the exposed surface. This chemical or ionising action of the rays (the same sort of action as enables them to cause an electroscope to leak) results in the production of ozone and of oxides of nitrogen: briefly, they have an oxidising action."

Bordier[2] shows that X-rays have an effect on the phenomena of osmosis, and the consequent interference with the molecular exchanges is followed by disturbances of nutrition and inflammation.

Now these marked changes in the tissues produced by the X-rays soon led to their adoption as a therapeutic agent, on the supposition that they ought to have an effect upon diseased cells. It has been found that tissues vary very much as to their sensibility to the action of the rays. Thus Holznecht,[3] describing their action on various tissues, divides them into classes as follows:—(1) Very sensible: Lymphoid tissue, the skin modified by psoriasis and mycosis fungoides. (2) Sensible: Skin modified by inflammation, acne, sycosis, lupus, and epitheliomatous tissue. (3) Moderately sensible: Healthy epidermis and its appendages. (4) Very little sensible: Connective tissue, vessels, etc.

One of the first diseases in which the rays were tried was that intractable disease **rodent ulcer**, and perhaps it is in this that the treatment has achieved one of its greatest successes, some cases being cured in a remarkably short space of time. Mayou[4] has examined microscopical sections of rodent ulcer

[1] Lectures to Medical Practitioners, *Ibid.*, v. 205.

[2] *Med. Electrol. and Radiol.*, vii. 72.

[3] *Arch. d'Electric. méd.*, Jan. 10, 25, 1905; abstract in *Med. Electrol. and Radiol.*, vi. 49.

[4] *Tr. Path. Soc. Lond.*, 1903, liv. 229.

during and after healing, and finds that "if a section of rodent ulcer be examined after it has begun to react to X-rays the clumps of epithelial cells which make up the growth are found surrounded by enormous numbers of leucocytes. We know it is the duty of these leucocytes to remove all irritating substances from the part by breaking them up and carrying them away; or, failing that, to encapsule them with fibrous tissue, shut off their nutrition, and so prevent their spreading. In a section of rodent ulcer which has nearly healed we find a mass of granulation tissue organising with healthy epithelium growing in from the edge. Scattered here and there are epithelial cells in various stages of degeneration; the degeneration seems to take place from without inwards. Towards the centre of the clump of epithelial cells the cells become vacuolated, the granules in the cells increase in numbers, collect around the cell-wall, and seem in places to be making their way through it till they quite disappear."

In my own experience some cases have done remarkably well and healed up quickly, but in others I must confess the ulceration has proved most obstinate, and I cannot help thinking that the cases which do well are the ones which I should call the dry variety, in which there is little or no secretion; but the moist variety, in which there is a copious secretion forming crusts, does not do so well: they heal up to a certain point, and then break down again and become as bad as ever. This result may be explained when we remember that serum and serous effusions are very opaque to the rays, as shown by the shadow thrown on the fluorescent screen by serous effusion in cases of pleurisy, consequently it is more difficult for the rays to reach the diseased cells, having first to penetrate the layer of serum before reaching the diseased structures.

It was fondly hoped in the first enthusiasm that was set up when the penetrating rays and their destructive action on the tissues of the body were discovered that at last a cure for **cancer** had been discovered; but, alas! although the rays have a very marked effect on the superficial manifestations of malignant disease, yet at present, at any rate, disease in the deeper structures of the body is beyond their influence. Still there can be no doubt that the X-rays are a most valuable form of treatment in inoperable carcinoma. Superficial nodules and ulcers will heal up under their influence, and enlarged glands will sometimes disappear, and, if for nothing else, they ought to be used for the very marked relief they give to the pain of malignant disease. It is sometimes amazing the relief thus afforded, especially in cases where a nerve is implicated either in the scar where the secondary growth has occurred, or on the surface of the ulcer, where the rays can readily reach it.

Never shall I forget one poor woman at the Infirmary with cancer *en cuirasse*, who continued to come for a dose of the X-rays as long as she could crawl simply for the relief she got from the excessive pain of the disease. The relief thus afforded is due most probably to the destructive action of the rays on the lecithin of the nerve tissue, as I have before mentioned.

Many observers have examined healed carcinomatous growths microscopically, and they all agree that the cells are primarily affected, and the growth is replaced by a dense formation of connective tissue. Thus Pusey and Caldwell[1] state: "The first changes occur in the cells at the periphery of each nest of carcinoma. Later the same process involves all the mass, invading in succession the cells from the periphery to the centre. These cells are found in various stages of destruction, or, if such it may be called, necrosis. The term necrosis, however, as ordinarily used, does not accurately describe the process as it occurs under X-rays. In the ordinary form of necrosis the cells retained more or less completely their form, and the first change induced is in the nuclei, which fail to take the basic stain, while the tissues in general take a diffused acid and basic stain, apparently a mixture of the two. On the other hand, in the tissue breaking down under X-rays, the cells and nuclei lose their outline, the chromatin of the nuclei appears to become spread out and mix with the protoplasm of the cells, where it is frequently seen as streaks or more or less irregular areas, staining a rather bright blue, and resembling to a certain extent mucoid degeneration. . . . The blood vessels, especially the small ones, which are in close relation to the tumour, show

[1] *Op. cit.*, p. 266.

marked endarteritis, completely occluding the lumina of the vessels. Around the occluded blood-vessels the breaking down of tumour cells is decidedly more pronounced than at any other place, even at the periphery of the cell nests. The tumour cells gradually disappear by a process which appears to be some form of cytolysis, which is then followed by their absorption."

In the final stage, "when the skin of the scar which replaced a former cancerous ulceration was examined, the carcinomatous tissue was replaced by a dense layer of healthy active tissue, whose fibres present a strikingly regular appearance." Enlarged glands in malignant disease sometimes disappear under the rays, and although they may appear enlarged or hardened, still this may be due to calcification of the gland and not to cancerous deposit. Thus Higham Cooper[1] quotes a case of recurrent cancer in which a supra-clavicular gland remained of a stony hardness, but when this gland came to be examined *post-mortem* (the patient having died of secondary deposit in the liver) it was found to be calcified and *not* malignant. Again, Knox[2] gives a detailed account of a case he had rayed in which he had specially examined the glands. In one gland which had been extensively rayed "the gland tissue was entirely replaced by a new growth which consisted of a fibrous stroma, in which existed large spaces blocked by epithelial cells. The growth was an epithelioma, in which the cells and nuclei were small and shrunken. Fibrous stroma appeared to be in excess, involving the areas of new growth, causing degeneration of the cells." Whole areas stained badly as regards fibrous stroma and cells. In a second gland, which became enlarged subsequently to the first, not nearly so much fibrous tissue was found, and the reaction to staining was more marked, both as regards fibrous tissue and cancer cells, and the latter were in larger groups and appeared more active. A third gland still more recently enlarged showed "columns of densely-packed malignant epithelial cells"; so that it seems that the X-rays have a very marked action on glands secondarily enlarged in malignant disease, the diseased cells being destroyed, and the gland tissue being replaced by a new growth of fibrous tissue.

[1] *Loc. cit.*, p. 5. [2] *Lancet*, 1906, i. 1755.

Wonderful cases of symptomatic cure have been reported, as for instance[1] a patient, aged 63, who had her right breast removed in 1899. Recurrence took place in the scar two years afterwards, when there was a thickened mass in the scar adherent to the ribs and skin, with a large ulcer 2 in. by 1½ in. with hard edges and a foul irregular base, the axillary glands were slightly enlarged, this being the condition when the X-ray treatment was commenced in November, 1901. After six weeks the ulcer was noticeably cleaner, and rapidly became superficial and healed. At the same time the hard mass decreased in size and became movable on the ribs, the gland in the axilla rapidly disappeared, and in January, 1902, there was only a slight thickening to be felt, and the scar was soft and healthy. In August, 1903, that is four years after the commencement of treatment, the patient was in excellent condition, the scar was soft and quite healthy, and there was no thickening to be felt.

I cannot point to any such successful case in my own practice, but a typical sort of case one gets is where in shorter or longer time after excision of the breast the scar and its neighbourhood becomes studded with nodules quite inoperable, and it is thought worth while to see what the rays will do. After a few exposures, as soon as a slight dermatitis is established, these nodules, invariably I may say, disappear, and the scar becomes perfectly smooth and healthy-looking; enlarged glands too subside and may disappear. The patient goes on quite well for a time, and then after a longer or shorter interval evidence of cancer of the lung, or of secondary deposit elsewhere makes its appearance, but I am certainly under the impression that this fatal result is postponed considerably by the treatment, the rays seeming to have a retarding influence over the progress of the disease, and the secondary disease does not ensue so rapidly when the patient is under treatment as it does when she is not.

Lyster[2] admirably sums up the present position of X-ray treatment of malignant disease thus: "X-ray treatment of malignant disease should never be attempted when an operation

[1] *Arch. Middlesex Hosp.*, 1904, ii. 141. [2] *Ibid.*, p. 142.

is possible, but in those cases where no operation is possible pain can be relieved, and in cases that are local and superficial the treatment may be considered hopeful, though no hope should be definitely held out of the growth vanishing. The word 'cure' should never be employed."

With this I cordially agree, but I still think that every patient suffering from inoperable carcinoma ought to have the benefit of X-ray treatment for the following reasons: (1) Because of the relief from pain; (2) because of the removal of local disease, which causes such mental relief; (3) because of the prolongation of life from retardation of the disease; and (4) on the off-chance that with the removal of the local mischief there may be no recurrence, as witnessed by the cases of apparent cure which have been reported.

In **sarcoma** the rays undoubtedly have a very marked effect, at any rate for a time. I have seen a mass in the scrotum as big as a turkey's egg, recurring after excision of the testis for sarcoma, entirely disappear, but in this case unfortunately large deposits subsequently occurred in the abdomen, causing a fatal issue. Again, in a hospital patient a man had his leg amputated for sarcoma of the tibia in August, 1905, and recurrence occurred in the stump in the periosteum of the femur in December; further operation was not thought advisable, and he was sent for treatment. At the present time, ten months after, there is no further advance in the disease, and there is nothing to be felt beyond a slight thickening around the femur. Here, although this man cannot be said to be cured, yet undoubtedly there is a checking of the disease; for if the disease is so malignant as to recur five months after operation, surely one would have expected an enormous advance in its ravages ten months later, instead of which the patient is at the present time in excellent health, and there is at any rate no evidence of the disease advancing in the affected part.

Another disease in which the treatment by X-rays has lately attracted a great deal of attention is **leukæmia**, and a number of cases in which apparently great benefit has been received have been reported. The number of the leucocytes in the blood has been greatly redneed in the successful cases, and this result is probably due to their destruction by the action of the rays. It has been found that X-ray radiation has a very marked effect on the lymphocytes and the lymphoid tissues generally. Heineke[1] has rayed the spleen in small animals, with the result that on examining the spleen there was "an excessive increase in pigment, disappearance of follicles, and destruction of the splenic pulp." In most of the cases treated there are symptoms of toxæmia, as shown by high temperature and other toxic symptoms, and in a few cases sudden death has occurred. Now if the leucocytes are destroyed by the rays, it is not to be wondered at that the circulation of effete material in the blood-stream should set up toxic symptoms, or even be the cause of sudden death; also, if the X-rays have such a destructive action on the spleen as Heineke asserts, it seems to me that to destroy the follicles of this gland and thus put out of action more or less the largest of the blood-forming organs of the body, requires a great deal of consideration; and on these grounds, if the treatment is to be used at all, very short exposures should be used, and the patient should be most carefully watched.

Perhaps the disease in which X-rays have been most extensively used is **lupus**, and here the treatment has perhaps achieved some of its greatest triumphs. The localised patches of lupus yield very readily as a rule to the treatment, but the more extensive the disease is the more difficult it seems to cope with it: even these cases yield in time, but only after prolonged treatment. Prolonged treatment in these cases is not peculiar to the X-rays, for the Finsen light, which gives such excellent results, requires to be applied for months or even years; and I have known cases where a patient has been treated with the Volkmann's spoon at the hands of the surgeon, and has had to undergo operation not once nor twice but several times, such scrapings extending over many months before a cure resulted.

The beneficial results in lupus are produced mainly by the leucocytosis set up, although the rays have a destructive effect

[1] *München. med. Wchnschr.*, 1904, li. 785; abstract in *Med. Electrol. and Radiol.*, July, 1904.

on the lowly-organised cells of the diseased part as well. It is now universally accepted that the rays have no bactericidal action. Grouven[1] describes the course of healing of lupus as follows: "Hyperæmia, leading to increased diapedesis of leucocytes, first at the periphery of the nodules, pressing on into the interior, changing into spindle cells and new connective tissue elements. The cells of the nodules undergo degeneration and absorption, and are replaced by connective tissue."

Lupus is a disease which seems to be most favourably influenced by opsonic treatment. It is reported that by observing the opsonic index of patients it can be foretold which cases are likely to react well to X-rays and which are not. Patients with a low opsonic index receive no benefit from the treatment, but as soon as the index is raised reaction occurs and healing rapidly follows,

In all these grave diseases which I have been discussing the rays have been called on to do what other methods of treatment have failed to accomplish, and to a certain extent they have achieved their purpose, but I think it is not sufficiently appreciated what a valuable means of treatment lies ready to the practitioner's hand in the X-rays in some minor diseases, especially in various forms of **skin disease.** Take for example psoriasis. In this disease it is wonderful to see the effect of the rays: a few applications of quite short duration to a patch of psoriasis will clear it up, leaving healthy skin behind, and this too without setting up any inflammation. And that the improvement brought about is in no way due to any method of treatment that is being used at the same time is shown by its being the patches that are rayed, and those patches alone, which are cured, patches in other parts of the body being unaffected. Thus, if the arms are treated they get well, but the legs, which may be equally bad, remain in the same state as before.

Of course one cannot expect the rays to remove the special dyscrasia which produces the disease, but that its local manifestations can be removed there is no doubt. Here again I cannot help thinking that why the rays have such a marked

[1] Quoted by Pusey and Caldwell, *op. cit.*, p. 263.

effect in this disease is because the rash is of a dry character, and the absence of serum on the surface enables the rays to reach the diseased structures so much more easily. All chronic hypertrophies of the skin are favourably affected, *e.g.* patches of dry eczema, lupoid patches, etc., disappearing as a rule after a few applications.

It is in the treatment of **diseases of the hair** that X-rays find one of their widest fields of usefulness. Here their action is, I may say, a mechanical one. The slight dermatitis which one aims at setting up causes a certain amount of inflammation in the hair follicles, which causes the hair to be shed with its accompanying fungus, allowing the parasitic remedies employed to be applied direct to the cause of the disease. The rays have to be applied with care, because it is quite possible to set up so much inflammation as to cause destruction of the hair follicles, and consequently cause permanent baldness, but with the rays carefully applied, what method of treatment can vie with them in such intractable complaints as sycosis and favus? Here as soon as the depilatory effect is produced the disease is speedily cured. Again, thanks to the labours of Sabouraud, the X-rays bid fair to revolutionise, if they have not already done so, the treatment of tinea tonsurans. By means of Sabouraud's pastilles or some form of radiometer by which the activity of the vacuum tube can be measured, a dose of rays is applied to the diseased patch, and in one or more applications depilation is effected without any pain, and much more effectually than by epilating with the forceps. In some of the London hospitals I have seen an apparatus which, by means of a radiometer to show the activity of the tube, and knowing by various instruments the strength and volume of the electric current passing through the tube, an exact knowledge of the amount of X-rays being used is ascertained. Accordingly, the physician in attendance orders how many units of X-ray radiation are to be applied, a clock is set to that figure, and the apparatus is turned on; as soon as the dose is administered the clock automatically switches off the current, and the patient is sent away with orders to report himself again in a month. Epilation is usually effected in a few days' time, but if this does not occur it is dangerous to apply the rays again at once, and the patient is told not to report himself for a month, when if necessary a second dose is administered.

Unfortunately it is not given to every hospital to have unlimited funds at its disposal for instruments of this kind which are more or less on their trial, but if such instruments can be perfected and shown to be absolutely reliable, every hospital in the country ought to be provided with one, and when it once became known that the means had been devised for curing ringworm speedily, it is not too much to hope that parents will bring their children for treatment much earlier than they do at present; and, also, is it too Utopian to suggest that in the not very far distant future tinea tonsurans may become, though not unknown, at any rate a much rarer disease than it is now.

As far as I have seen, very good results may be obtained by working carefully without any of these elaborate instruments, by using a tube of moderate hardness, giving five minutes exposure twice a week and stopping when there is a trace of redness on the skin. As soon as this latter occurs the hairs come out readily, and very speedily the patch is cured.

From facts I have brought before you in this brief résumé of X-ray therapeutics it is very evident that the rays have a very marked effect on the superficial tissues of the body, but as yet, with the means at our disposal, the deeper structures have escaped their remedial influence. It must, however, be remembered that Röntgen only made his discovery ten years ago, and that radio-therapy is therefore quite a young science. Improved methods of producing the rays are constantly being brought forward, and experience in their use is daily accumulating, so that with increased power in the operator's hands and enlarged experience in the use of this therapeutic agent, I think we may confidently look forward to a greatly-extended use of this valuable method of treatment in the near future.

